# Prenatal Vitamin D Levels in Maternal Sera and Offspring Specific Learning Disorders

**DOI:** 10.3390/nu13103321

**Published:** 2021-09-23

**Authors:** Bianca Arrhenius, Subina Upadhyaya, Susanna Hinkka-Yli-Salomäki, Alan S. Brown, Keely Cheslack-Postava, Hanna Öhman, Andre Sourander

**Affiliations:** 1 Department of Child Psychiatry, University of Turku, 20014 Turku, Finland; subina.upadhyaya@utu.fi (S.U.); sushys@utu.fi (S.H.-Y.-S.); 2 INVEST Research Flagship, University of Turku, 20014 Turku, Finland; 3 Department of Psychiatry, New York State Psychiatric Institute, Columbia University, New York, NY 10032, USA; asb11@cumc.columbia.edu (A.S.B.); kc2497@cumc.columbia.edu (K.C.-P.); 4 Department of Epidemiology, Columbia University Mailman School of Public Health, New York, NY 10032, USA; 5 Faculty of Medicine, University of Oulu, 90014 Oulu, Finland; hanna.ohman@ppshp.fi; 6 Biobank Borealis of Northern Finland, Oulu University Hospital, 90014 Oulu, Finland; 7 Department of Child Psychiatry, Turku University Hospital, 20521 Turku, Finland

**Keywords:** learning disorder, reading, writing, math, prenatal, maternal, vitamin D

## Abstract

Recent evidence has suggested potential harmful effects of vitamin D deficiency during pregnancy on offspring brain development, for example, elevated risks for neuropsychiatric disorders. Findings on general cognition and academic achievement are mixed, and no studies have examined the effect of prenatal 25-hydroxyvitamin D (25(OH)D) levels on diagnosed specific learning disorders, which was the aim of this study. We examined a nested case–control sample from the source cohort of all singleton-born children in Finland between 1996 and 1997 (*n* = 115,730). A total of 1607 cases with specific learning disorders (mean age at diagnosis: 9.9 years) and 1607 matched controls were identified from Finnish nationwide registers. Maternal 25(OH)D levels were analyzed from serum samples collected during the first trimester of pregnancy and stored in a national biobank. Conditional logistic regression was used to test the association between maternal 25(OH)D and offspring specific learning disorders. There were no significant associations between maternal 25(OH)D levels and specific learning disorders when vitamin D was examined as a log-transformed continuous variable (adjusted OR 0.98, 95% CI 0.82–1.18, *p* = 0.84) or as a categorical variable (25(OH)D < 30 nmol/L: adjusted OR 1.03, 95% CI 0.83–1.28, *p* = 0.77 compared to levels of >50 nmol/L), nor when it was divided into quintiles (adjusted OR for the lowest quintile 1.00, 95% CI 0.78–1.28, *p* = 0.99 compared to the highest quintile). This study found no association between low maternal 25(OH)D in early pregnancy and offspring specific learning disorders.

## 1. Introduction

Maternal vitamin D is essential for fetal growth and development, and prenatal exposure to vitamin D deficiency during a critical period of brain development may result in persistent functional alterations in the brain. Vitamin D receptors and vitamin D metabolizing enzymes are largely expressed in brain cells and tissues [1,2], where vitamin D regulates neurological functions [3]. Experimental animal studies have shown that vitamin D deficiency during gestation is associated with morphological changes in the brain [3,4]. In humans, maternal vitamin D deficiency has been associated with unfavorable pregnancy outcomes, including gestational diabetes [5], impaired offspring bone development [6], prematurity [7], and subsequent risk of delayed cognitive impairment and neuropsychiatric disorders [3]. A recent nationwide nested case–control study found that lower levels of prenatal vitamin D were associated with offspring autism spectrum disorders [8]. Furthermore, studies have shown significant associations between low prenatal vitamin D levels and offspring schizophrenia and attention deficit hyperactivity disorder (ADHD) [9,10]. However, no previous studies have examined the association between prenatal vitamin D levels and diagnosed specific learning disorders in offspring. Specific learning disorders refer to reading, writing, and arithmetic disorders, and they are common among children with prevalence estimates of 6–10% [11,12].

Although research on the association between measured maternal vitamin D and specific learning disorders is lacking, there are some studies on general cognitive and language development in children or adolescents. An Australian study [13] measured vitamin D from maternal sera in the 18th week of gestation and found an elevated risk of language impairment at ages 5 and 10 in offspring of vitamin D-deficient mothers. A Danish study [14] measured vitamin D levels from newborns and their intelligence quotient (IQ) at age 19 and discovered that the two lowest quintiles of vitamin D had slightly lower general IQ than those in the higher quintiles. A UK study including 422,512 children measured antenatal exposure to sunlight and found that the total amount of UVB light during pregnancy was inversely associated with the risk of a learning disability, with some evidence of a dose relationship [15]. The authors hypothesized the effect to be due to lower levels of vitamin D during periods with less sunlight. However, some other studies have not found associations between vitamin D and cognitive ability [16,17,18,19] or scholastic achievement [20] in school-aged children. Furthermore, a number of studies have examined developmental and cognitive outcomes in babies and toddlers with mixed findings [21,22,23]. The current evidence supports vitamin D supplementation during pregnancy to prevent gestational diabetes and low birthweight in offspring [24], but evidence is limited on the benefits of supplementation on cognitive outcomes in offspring [25]. The use of multiple micronutrient supplements during pregnancy has shown no beneficial effects on offspring cognitive outcomes [26].

To address the knowledge gap of studies on maternal vitamin D during pregnancy and offspring specific learning disorders, we conducted a nationwide nested case–control study based on Finnish national registers. The assessment of vitamin D during pregnancy was based on unique serum samples from mothers of children born 1996–1997 in Finland. The pregnancies included in the study took place before the national vitamin D food fortification policy was initiated in 2003 and the recommendations of vitamin D intake during pregnancy were increased in 2005 [27]. The aim was to study the association between vitamin D levels during early pregnancy and diagnosed specific learning disorders in offspring. Based on the findings of vitamin D deficiency and several neuropsychiatric disorders, as well as possible seasonality effects on learning disorders found in previous studies, we hypothesized that lower prenatal vitamin D levels would be associated with an increased risk of offspring learning disorders. This would have potential implications for preventive strategies, including vitamin D supplementation policies. Since specific learning disorders are so common, increased prevention would have a significant public health impact. Moreover, it might benefit less advantaged populations in particular, for example, immigrants and the socially disadvantaged, who may be affected by both vitamin D deficiency [28,29] and learning disorders [30,31].

## 2. Materials and Methods

This work is part of a national nested case–control study that includes all singleton live births in Finland from 1996 to 2007. For this study, we included a subset of children born in 1996–1997 with diagnoses available from the Care Register for Health Care (CRHC) by the end of 2012. The ethical approval for the study was provided by the Ethics Committee of the Hospital District of Southwest Finland, by the data protection authorities at the National Institute for Health and Welfare, and by the Institutional Review Board of the New York State Psychiatric Institute.

### 2.1. Finnish Maternity Cohort

The Finnish Maternity Cohort (FMC) includes two million maternal serum samples, which were collected mainly in the first trimesters of pregnancy (5th to 95th percentile: months 2–4 of pregnancy) from almost one million pregnancies. Informed consent was obtained for the collection of prenatal serum specimens at maternity clinics for routine screening of congenital infections. The remaining 1–3 mL of the sample from each mother was then stored at −25 °C in a protected biorepository at Biobank Borealis in Oulu, Finland, and made available for scientific research. The unique personal identification code, which is assigned to all Finnish residents, was used to link the FMC samples with other Finnish registers.

### 2.2. National Registers

The CRHC was used to identify all the cases, by detecting registered diagnoses of specific learning disorders by the end of 2012. In the CRHC, inpatient diagnoses from specialized services have been available since 1967 and outpatient diagnoses since 1998. The diagnoses in the CRHC are recorded according to the International Classification of Diseases (ICD) classification: ICD-8 from 1969 to 1986, ICD-9 from 1987 to 1995, and ICD-10 since 1996. The CRHC also provided information on parental psychopathology. The Finnish Maternity Birth Register (FMBR) was used to extract variables related to maternal health and pregnancy, delivery, and newborn health. The Digital and Population Data Services Agency (DVV, formerly known as the Finnish Population Register Centre) was used to identify the controls and to obtain information on the subjects’ parents. The DVV manages the demographic information of everyone living in Finland, which includes the name, personal identification code, address, native language, citizenship, family information, and date of birth and death.

### 2.3. Information on Cases and Controls

The cases were born between 1.1.1996 and 31.12.1997 and diagnosed with a specific learning disorder (ICD-10: F81.x) by 31.12.2012 in the CRHC. Cases with comorbid autism spectrum disorder (ASD, F84) and/or intellectual disability (ID, F70–79) were excluded. In Finland, learning disorders are usually diagnosed in publicly funded outpatient clinics of pediatric neurology, pediatrics, or child psychiatry. If learning disorders are suspected in schools or primary care, children are typically examined first by the school psychologist and then, if needed, referred to specialized services for diagnosis. The procedure includes standardized psychological tests and has been described previously [30].

In addition to the main outcome, which was any specific learning disorder diagnosis (F81.x), we also stratified cases into mutually exclusive subgroups of specific learning disorders. These included: reading disorder (F81.0); spelling disorder (F81.1); arithmetic disorder (F80.2); and a mixed group consisting of mixed scholastic disorders (F80.3), other scholastic disorders (F80.8), unspecified scholastic disorders (F80.9) and multiple diagnoses of reading, spelling, and arithmetic disorders (F81.0, F81.1, and F80.2). Furthermore, because of previous associations found between vitamin D deficiency and offspring ADHD, we stratified the specific learning disorder cases according to the presence of comorbid ADHD (F90.x).

The cases were matched with single controls who did not have a diagnosis of speech/language, learning, or coordination disorder (F80–83), ASD, or ID by date of birth (±30 days) and gender. Furthermore, the controls were born, alive, and residing in Finland at the time of the corresponding case’s diagnosis.

### 2.4. Maternal Serum 25(OH)D Assay

The measurement of maternal serum 25(OH)D was performed blind to case–control status using a chemiluminescence microparticle immunoassay (CMIA) by an Architect i2000SR automatic analyzer (Abbott Diagnostics). The procedure has previously been described in detail [10,32].

### 2.5. Covariates

Covariates that have been associated with maternal vitamin D levels and offspring learning disorders in previous studies were initially selected [24,28,29,33,34]. Information on maternal smoking, age, number of previous births, and socioeconomic status (SES), as well as offspring gestational age, birth weight, and Apgar score, were obtained from the FMBR, maternal immigrant status from the DVV, and the season and gestational week of blood draw from the FMC. Data on maternal and paternal psychopathology and history of maternal substance abuse were obtained from the CRHC. For the classification of the covariates, see Supplementary Table S1.

### 2.6. Statistical Analysis

First, we examined maternal vitamin D as a continuous variable, which was natural log transformed before the analyses due to a skewed distribution. Next, we categorized the maternal vitamin D levels into quintiles, with cutoff points that were based on the distribution of maternal vitamin D in the control group. Furthermore, maternal vitamin D was categorized based on clinical categories: (1) deficient (25(OH)D < 30 nmol/L), (2) insufficient (25(OH)D 30–49.9 nmol/L), and (3) sufficient levels of vitamin D (25(OH)D > 50 nmol/L) [35]. The highest quintile and the sufficient category served as reference groups. We used the continuous measure of maternal vitamin D as the exposure variable in the sensitivity analyses of specific learning disorder subgroups and ADHD comorbidity.

The associations between the covariates and (1) maternal 25(OH)D among controls and (2) specific learning disorders were tested with Student’s T- and F-tests for continuous covariate variables or Pearson chi-square tests for categorical variables. Covariates were included in the adjusted model if they were associated with both exposure and outcome at *p* < 0.1 [36]. A chi-square test was used to test the possible gender interaction for the association between continuous maternal vitamin D and specific learning disorders as well as the collinear association of maternal SES and smoking. We used conditional logistic regression for matched pairs to calculate odds ratios (ORs) with 95% confidence intervals (CIs). Statistical significance was based on *p* < 0.05 in all other analyses except for the covariate testing. SAS software was used to perform the statistical analyses (SAS 9.4, SAS Institute, Cary, NC, USA).

## 3. Results

Among all 115,730 singleton-born children in Finland between 1 January 1996 and 31 December 1997, 2174 children were diagnosed with a specific learning disorder in specialized health care by the end of 2012. Of these children, 81 (3.7%) had ID, 111 (5.1%) had ASD, and 11 (0.5%) had both ID and ASD and were therefore excluded. Of the remaining 1971 cases with a specific learning disorder, 1621 cases and the equal number of controls had a maternal serum sample available in the FMC collection. We further excluded 14 case–control pairs because of ID and/or ASD diagnoses among the controls. The final sample comprised 1607 cases and 1607 controls.

**Figure 1:**
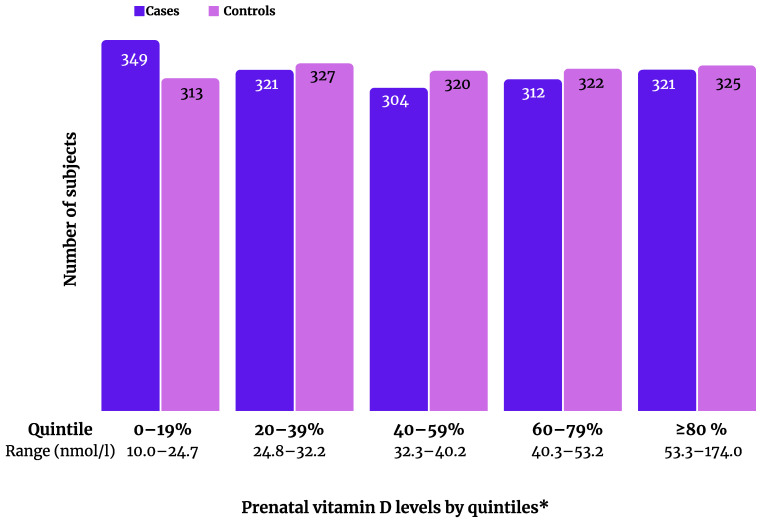
Distribution of maternal 25-hydroxyvitamin D levels in cases with specific learning disorders and matched controls. * Quintiles based on the distribution in controls.

The mean age at specific learning disorder diagnosis was 9.9 years (standard deviation, SD 2.9). Among the 1607 cases and controls, 1129 (70.3%) were male and 478 (29.7%) were female. The median maternal 25(OH)D level was 39.3 nmol/L (SD 18.0; range 10.8–146.8 nmol/L) for cases and 39.9 nmol/L (SD 17.9; range 10.0–174.0 nmol/L) for controls. The mean gestational week of maternal blood collection was 11.0 (SD 3.5) for cases and 10.6 (SD 3.1) for controls. Maternal gestational diabetes mellitus was reported in 4.79% of the case pregnancies and 2.61% of the control pregnancies, and pre-eclampsia was reported in 0.75% and 0.37%, respectively. The distribution of maternal 25(OH)D categorized into quintiles among cases and controls is presented in Figure 1.

Among the covariates, maternal age, smoking, and SES as well as offspring weight for gestational age were significantly associated with both vitamin D levels among the controls and specific learning disorder diagnosis (Supplementary Table S1). Because of the association between maternal smoking and SES (*p* <0.001, data not shown), we included only SES. When we adjusted with smoking instead of SES, or both variables, the results were similar (data not shown). The final adjusted model included maternal age, SES, and offspring weight for gestational age.

We found no significant associations between maternal vitamin D and offspring specific learning disorder in the logistic regression analyses when vitamin D was examined as a continuous log-transformed (aOR 0.98, 95% CI 0.82–1.18, *p* = 0.84) or categorical variable (25(OH)D <30 nmol/L: aOR 1.03, 95% CI 0.83–1.28, *p* = 0.77), nor when it was divided into quintiles (aOR for the lowest quintile 1.00, 95% CI 0.78–1.28, *p* = 0.99) (Table 1). The results did not differ in the crude and adjusted analyses (Table 1). Of note, as shown in Figure 1, the number of cases was slightly higher in the lowest quintile compared with the number of controls, but the difference was not statistically significant in the unadjusted or adjusted analyses (Table 1). In addition, we performed analyses with vitamin D levels divided into deciles, and there were also no statistically significant findings in the decile groups (data not shown). Moreover, there was no interaction by gender for the association between continuous log-transformed maternal vitamin D and offspring specific learning disorder (*p* for interaction = 0.61, aOR 0.98 (95% CI 0.79–1.23, *p* = 0.89) for boys and aOR 1.01 (95% CI 0.70–1.44, *p* = 0.97) for girls).

In the sensitivity analyses, associations between continuous maternal vitamin D and specific learning disorder subgroups, namely, reading, spelling, and arithmetic disorders, or mixed specific learning disorders were similar across groups (Table 2). However, the low number of subjects in the spelling and arithmetic disorder groups limited the conclusions for these subgroups. Among the 1607 cases, 358 (22.3%) had ADHD, while the frequency among controls was 37 (2.3%). Comorbid ADHD did not affect the association between continuous maternal vitamin D and specific learning disorders (aOR 1.06 (95% CI 0.86–1.31, *p* = 0.59) for the group without comorbid ADHD and aOR 0.76 (95% CI 0.50–1.16, *p* = 0.21) for the group with comorbid ADHD.

## 4. Discussion

This is the first study to examine the association between maternal vitamin D levels in prenatal sera and diagnosed specific learning disorders in offspring. We found no significant association between maternal vitamin D and offspring specific learning disorders, but the number of cases was slightly higher compared with the number of controls in the lowest quintile. The findings are in line with some previous studies that have examined maternal serum vitamin D and learning-related outcomes, such as academic achievement or IQ in school-aged children or adolescents [16,17,20]. In contrast, the Australian study that examined language impairment in 5- and 10-year old children [13] and the Danish study [14] that looked at cognitive abilities in adolescents reported positive associations. Differing outcome variables and timing of the vitamin D measurement from maternal sera (early or late pregnancy, cord blood at birth) might explain the heterogeneous findings across studies.

The findings from the present study do not support our hypothesis that prenatal vitamin D deficiency would be an etiological factor for specific learning disorders. Some previous studies have reported positive associations for neuropsychiatric disorders, including ASD, ADHD, and schizophrenia [8,9,10]. Animal studies have found altered brain morphology, physiology, and gene expression in rodent offspring prenatally deprived of vitamin D. Symptoms have manifested as behavioral dysfunction, increased locomotion, and anxiety, as well as more subtle effects on learning and memory [37]. Additionally, rodent studies have suggested an effect of low prenatal vitamin D on dopaminergic systems [4], which might partly explain why disorders with possible abnormal dopaminergic signaling, such as schizophrenia and ASD, have been associated with prenatal vitamin D deficiency, while in the present study, specific learning disorders were not.

The strengths of this study include the large, randomly selected samples from nationwide data sources and maternal serum samples during pregnancy, which were collected prospectively. Another strength is the use of a uniform diagnostic system (ICD). The specific learning disorder diagnoses from the CRHC are verified by a physician, in contrast to many other studies in which the diagnoses of specific learning disorders have relied on parent report.

However, the register-based diagnoses from specialized services also pose a limitation, as children with milder learning disorders are typically not referred to these services. Therefore, conclusions are limited to the more impending cases of specific learning disorders. Further, the number of cases with arithmetic disorders and spelling disorders, but no other specific learning disorder diagnoses, was small compared to the whole sample. Therefore, the associations were driven predominantly by the cases with multiple or mixed specific learning disorders, for example, reading disorder in combination with arithmetic disorder, and the cases with only reading disorders.

It is also worth noting that the serum samples in the study were collected in the first or early second trimester of the pregnancy only, which limits our conclusions of the possible effect of maternal vitamin D deficiency during later phases of pregnancy on offspring specific learning disorders. However, systematic reviews [21,23] have concluded that studies were more likely to find positive associations between low maternal vitamin D levels and adverse cognitive outcomes if the serum samples were collected in early or mid-pregnancy. Furthermore, studies that have measured serum vitamin D at multiple time points in pregnancy have found correlated measures over the course of pregnancy [19,38].

## 5. Conclusions

Previous studies have suggested prenatal vitamin D as an important factor for many developmental outcomes in offspring. Particularly, prenatal vitamin D deficiency has been associated with neuropsychiatric outcomes such as ASD, ADHD, and schizophrenia. However, this study found no association between prenatal vitamin D deficiency and offspring specific learning disorders.

## Figures and Tables

**Table 1:** Association between maternal serum vitamin D and specific learning disorders in offspring.

Maternal Vitamin D (nmol/L)	Cases *n* = 1607	Controls *n* = 1607	Crude OR (95% CI)	*p*	Adjusted OR ^1^ (95% CI)	*p*
Continuous	Mean (SD)	Mean (SD)				
	39.3 (18.0)	39.9 (17.9)	0.88 (0.74–1.06)	0.17	0.98 (0.82–1.18)	0.84
Quintiles ^2^	Frequency (%)	SFrequency (%)				
0–19%	349 (21.7)	313 (19.5)	1.16 (0.91–1.47)	0.23	1.00 (0.78–1.28)	0.99
20–39%	321 (20.0)	327 (20.3)	1.01 (0.81–1.28)	0.90	0.95 (0.75–1.20)	0.67
40–59%	304 (18.9)	320 (19.9)	0.97 (0.77–1.22)	0.81	0.94 (0.74–1.19)	0.60
60–79%	312 (19.4)	322 (20.0)	0.99 (0.79–1.23)	0.89	0.92 (0.73–1.16)	0.50
≥80%	321 (20.0)	325 (20.2)	Reference		Reference	
Categorical	Frequency (%)	Frequency (%)				
<30	598 (37.2)	541 (33.7)	1.15 (0.94–1.42)	0.17	1.03 (0.83–1.28)	0.77
30-<50	632 (39.3)	685 (42.6)	0.95 (0.78–1.14)	0.55	0.91 (0.75–1.10)	0.33
≥50	377 (23.5)	381 (23.7)	Reference		Reference	

OR: odds ratio. ^1^ Adjusted for maternal age, socioeconomic status, and offspring weight for gestational age. For socioeconomic status, the missing category was included as a separate level. Ten cases and eight controls were omitted from the adjusted analyses because of missing data. ^2^ Distribution based on vitamin D levels in controls.

**Table 2:** Odds ratios and 95% confidence intervals of the association between continuous maternal serum vitamin D and subtypes of specific learning disorders.

Specific Learning Disorder	Cases N = 1607	Maternal Vitamin D (nmol/L) Mean (SD)		Crude OR (95% CI)	*p*	Adjusted OR ^1^ (95% CI)	*p*
	Frequency (%)	Cases	Controls				
Reading disorder only	304 (18.9)	38.7 (16.3)	38.7 (16.0)	0.98 (0.64–1.48)	0.90	1.14 (0.74–1.77)	0.55
Spelling disorder only	39 (2.4)	39.8 (20.4)	40.0 (17.6)	0.87 (0.28–2.7)	0.80	1.40 (0.33–6.00)	0.65
Arithmetic disorder only	22 (1.4)	43.6 (20.9)	38.9 (17.6)	3.2 (0.44–23.3)	0.25	7.6 (0.33–174.2)	0.21
Mixed or multiple specific learning disorders	1242 (77.3)	39.3 (18.2)	40.2 (18.4)	0.85 (0.70–1.04)	0.12	0.93 (0.75–1.14)	0.47

CI, confidence interval. OR, odds ratio. ^1^ Adjusted for maternal age, socioeconomic status, and offspring birthweight for gestational age. Missing data for covariates in the adjusted analyses: reading disorder only: 3 controls, 2 cases; mixed or multiple learning disorders: 7 controls, 6 cases. Categories according to the International Classification of Diseases, 10th edition: Reading disorder only: F81.0 diagnosis, no other F81.x diagnoses. Spelling disorder only: F81.1 diagnosis, no other F81.x diagnoses. Arithmetic disorder only: F81.2 diagnosis, no other F81.x diagnoses. Mixed or multiple learning disorders: F81.3, F81.8, F81.9 diagnosis and/or two or more diagnoses of F81.0. F81.1 and F81.2.

## Data Availability

Deidentified individual participant data will not be made available due to ethical restrictions. Summary level data can be obtained from the corresponding author upon reasonable request.

## Supplementary Materials

The following are available online at https://www.mdpi.com/article/10.3390/nu13103321/s1, Table S1: Relationship between covariates and maternal serum vitamin D among controls, and covariates, and specific learning disorder diagnosis in case and control subjects.
