# Supplementary material for: Prenatal Vitamin D Levels in Maternal Sera and Offspring Specific Learning Disorders
Source: Nutrients. 2021 Sep 23;13(10):3321. doi: 10.3390/nu13103321 (PMC8539854; doi:10.3390/nu13103321)
Supplement: Supplementary file 1 [file nutrients-13-03321-s001.zip › nutrients-1366953-supplementary.pdf]

Supplementary Table S1: Relationship between covariates and maternal serum vitamin D among controls, and covariates and specific learning disorder diagnosis in case and control subjects.

	Covariates and vitamin D (nmol/L) among controls				Covariates and specific learning disorder		
	Deficient<br><30<br>N=541	Insufficient<br>30-<50<br>N=685	Sufficient<br>>50<br>N=381		Cases<br><br>N=1,607	Controls<br><br>N=1,607	
**Categorical variables**	**Frequency (%)**	**Frequency (%)**	**Frequency (%)**	**x^2 test<br>P-value**	**Frequency (%)**	**Frequency (%)**	**x^2 test<br>P-value**
**Maternal smoking**							
No	416 (79.1)	564 (83.9)	327 (87.0)	0.006	1,194 (76.5)	1,307 (83.0)	<0.001
Yes	110 (20.9)	108 (16.1)	49 (13.0)		366 (23.5)	267 (17.0)	
**Previous births**							
0	226 (41.9)	268 (39.2)	146 (38.6)	0.53	598 (37.3)	640 (40.0)	0.12
≥ 1	314 (58.1)	416 (60.8)	232 (61.4)		1,006 (62.7)	962 (60.0)	
**Maternal psychiatric history**[a]							
No	473 (87.4)	604 (88.2)	339 (89.0)	0.77	1,272 (79.2)	1,416 (88.1)	<0.001
Yes	68 (12.6)	81 (11.8)	42 (11.0)		335 (20.8)	191 (11.9)	

**Paternal psychiatric history**[b]							
No	461 (86.0)	600 (88.4)	336 (88.2)	0.42	1,259 (79.9)	1,397 (87.5)	<0.001
Yes	75 (14.0)	79 (11.6)	45 (11.8)		316 (20.1)	199 (12.5)	
**Maternal SES**							
Upper white collar	66 (12.9)	115 (17.4)	55 (15.0)	0.03	129 (8.5)	236 (15.3)	<0.001
Lower white collar	228 (44.7)	319 (48.2)	189 (51.6)		664 (43.6)	736 (47.9)	
Blue collar	123 (24.1)	124 (18.7)	61 (16.7)		417 (27.4)	308 (20.0)	
Others	93 (18.2)	104 (15.7)	61 (16.7)		313 (20.6)	258 (16.8)	
**History of maternal substance abuse**[c]							
No	534 (98.7)	668 (97.5)	375 (98.4)	0.28	1,534 (95.5)	1,577 (98.1)	<0.001
Yes	7 (1.3)	17 (2.5)	6 (1.6)		73 (4.5)	30 (1.9)	
**Maternal immigration status**							
No	529 (97.8)	678 (99.0)	375 (98.4)	0.24	1,607 (100.0)	1,582 (98.4)	<0.001
Yes	12 (2.2)	7 (1.0)	6 (1.6)		0 (0.0)	25 (1.6)	
**Weight for gestational age**							
<-2 SD	24 (4.5)	19 (2.8)	5 (1.3)	0.07	100 (6.3)	48 (3.0)	<0.001
-2 SD to +2 SD	497 (92.4)	642 (93.9)	355 (94.2)		1,439 (90.1)	1,494 (93.4)	

>+2 SD	17 (3.2)	23 (3.4)	17 (4.5)		58 (3.6)	57 (3.6)	
**Gestational age**							
<37 weeks	21 (3.9)	27 (4.0)	14 (3.7)	0.98	113 (7.1)	62 (3.9)	<0.001
≥37 weeks	519 (96.1)	657 (96.0)	363 (96.3)		1,485 (92.9)	1,539 (96.1)	
**Season of blood draw**							
Spring	226 (41.8)	182 (26.6)	39 (10.2)	<0.001	442 (27.5)	447 (27.8)	0.94
Summer	28 (5.2)	152 (22.2)	187 (49.1)		357 (22.2)	367 (22.8)	
Autumn	83 (15.3)	175 (25.6)	115 (30.2)		385 (24.0)	373 (23.2)	
Winter	204 (37.7)	176 (25.7)	40 (10.5)		423 (26.3)	420 (26.1)	
**Apgar Score**							
0–6	25 (4.7)	22 (3.2)	14 (3.7)	0.43	80 (5.0)	61 (3.8)	0.10
7–10	512 (95.3)	662 (96.8)	363 (96.3)		1,512 (95.0)	1,537 (96.2)	
**Continuous variables**	**Least squares means (SD)**			**F-test P-value**	**Mean (SD)**	**Mean (SD)**	**T-test P-value**
**Maternal age (years)**	28.7 (5.1)	29.6 (5.1)	29.6 (5.1)	0.003	28.8 (5.7)	29.3 (5.1)	0.005
**Gestational week of blood draw**	10.7 (3.1)	10.5 (3.1)	10.5 (3.1)	0.31	11.0 (3.1)	10.6 (3.5)	<0.001

[a] ICD-8 (291-308), ICD-9 (291-316) or ICD-10 (F10-99, excluding maternal substance abuse diagnosis, [b] ICD-8 (291-308), ICD-9 (291-316) or ICD-10 (F10-99); [c] ICD-8 (291, 303, 304), ICD-9 (291, 292, 303,304,305) or ICD-10 (F10-19). Abbreviations: SES, socioeconomic status. SD, standard deviation.

Missing data: Maternal smoking 80 subjects; Maternal SES 153 subjects; Previous births 8 subjects; Paternal psychiatric history 43 subjects; Gestational age 15 subjects; Weight for gestational age 18 subjects; Apgar score 24 subjects.
